# The Human Gene Mutation Database: building a comprehensive mutation repository for clinical and molecular genetics, diagnostic testing and personalized genomic medicine

**DOI:** 10.1007/s00439-013-1358-4

**Published:** 2013-09-28

**Authors:** Peter D. Stenson, Matthew Mort, Edward V. Ball, Katy Shaw, Andrew D. Phillips, David N. Cooper

**Affiliations:** Institute of Medical Genetics, School of Medicine, Cardiff University, Heath Park, Cardiff, CF14 4XN UK

**Keywords:** Unify Medical Language System, Mutation Data, Human Gene Mutation Database, Human Phenotype Ontology, Variant Call Format

## Abstract

The Human Gene Mutation Database (HGMD^®^) is a comprehensive collection of germline mutations in nuclear genes that underlie, or are associated with, human inherited disease. By June 2013, the database contained over 141,000 different lesions detected in over 5,700 different genes, with new mutation entries currently accumulating at a rate exceeding 10,000 per annum. HGMD was originally established in 1996 for the scientific study of mutational mechanisms in human genes. However, it has since acquired a much broader utility as a central unified disease-oriented mutation repository utilized by human molecular geneticists, genome scientists, molecular biologists, clinicians and genetic counsellors as well as by those specializing in biopharmaceuticals, bioinformatics and personalized genomics. The public version of HGMD (https://www.hgmd.cf.ac.uk) is freely available to registered users from academic institutions/non-profit organizations whilst the subscription version (HGMD Professional) is available to academic, clinical and commercial users under license via BIOBASE GmbH.

## Introduction

The Human Gene Mutation Database (HGMD^**®**^) represents an attempt to collate all known gene lesions responsible for causing human inherited disease together with disease-associated/functional polymorphisms that have been published in the peer-reviewed literature. These data comprise single base-pair substitutions in coding, regulatory and splicing-relevant (both intronic and exonic) regions of human nuclear genes, as well as micro-deletions and micro-insertions, combined micro-insertions/micro-deletions (indels) of 20 bp or less, repeat variations, gross lesions (deletions, insertions and duplications of greater than 20 bp, up to and including a single characterized gene or group of contiguous genes that are directly involved in the aetiology of the disease/phenotype) and complex rearrangements (including inversions, translocations and complex indels). Mutation data are summarized in Table 1.

**Table 1:** Numbers of different mutations by mutation type present in HGMD Professional 2013.2 and the publicly available version of the database (June 28th 2013)

Mutation type	Total numbers of mutations		
	HGMD Professional	With chromosomal coordinates	Publicly available
Missense substitutions	62,368	61,845	44,933
Nonsense substitutions	15,781	15,574	11,306
Splicing substitutions	13,030	12,538	9,467
Regulatory substitutions	2,751	2,713	1,753
Micro-deletions ≤ 20 bp	21,681	21,134	15,796
Micro-insertions ≤20 bp	8,994	8,721	6,494
Micro-indels ≤20 bp	2,083	2,004	1,459
Gross deletions >20 bp	10,267	0	6,156
Gross insertions/duplications >20 bp	2,376	0	1,253
Complex rearrangements	1,409	0	946
Repeat variations	421	0	305
Totals	141,161	124,529	99,868

HGMD does not include either somatic or mitochondrial mutations, which are well covered by COSMIC (Forbes et al. 2011) and MitoMap (Ruiz-Pesini et al. 2007), respectively. HGMD also does not attempt to provide comprehensive coverage of pharmacological variants (except for those variants where evidence supporting a functional impairment has been provided); such variants are covered by PharmGKB (Thorn et al. 2010). Finally, HGMD is not a general genetic variation database; users interested in this type of variant should visit dbSNP (Sherry et al. 2001) or the Exome Variant Server (http://evs.gs.washington.edu/EVS/).

HGMD was originally established for the scientific study of mutational mechanisms in human genes causing inherited disease (Cooper et al. 2010), but has since acquired a much broader utility as a central unified repository for germ-line disease-related functional variation. It is now routinely accessed and utilized by next generation sequencing (NGS) project researchers, human molecular geneticists, molecular biologists, clinicians and genetic counsellors as well as by those specializing in biopharmaceuticals, bioinformatics and personalized genomics.

HGMD is available in two versions: one public, one obtainable by subscription. The public version of HGMD (http://www.hgmd.cf.ac.uk) is freely available to registered users from academic institutions/non-profit organizations. This version is, however, maintained in a basic form that is only updated twice per annum, is permanently 3 years out of date, and does not contain any of the additional annotations or extra features present in HGMD Professional (such as GRCh37/hg19 genomic chromosomal coordinates, HGVS nomenclature and additional literature references, see Table 2). The Professional version is available to both commercial and academic/non-profit users via subscription from BIOBASE GmbH (http://www.biobase-international.com).

**Table 2:** Differences between HGMD Professional and HGMD Public

	HGMD Professional	HGMD Public
Up-to-date mutation data	✓	✗
Curator comments	✓	✓
Quarterly updates^+^	✓	✗
Gene-oriented search	✓	✓
Mutation-oriented search	✓	✗
Reference-oriented search	✓	✗
Batch search mode	✓	✗
Chromosomal coordinates	✓	✗
HGVS nomenclature	✓	✗
Additional literature references	✓	✗
Tracked variant history	✓	✗
dbSNP identifiers	✓	✗
Enhanced search options	✓	✗
Advanced search features	✓	✗
Disease ontology terms*	✓	✗
Data in VCF*	✓	✗
Downloadable version	✓	✗

^+^HGMD Public is updated on a 6-monthly basis

* Download customers only

## Acquisition of mutation data

All HGMD mutation data are manually curated from the scientific literature. Identification of relevant literature reports is carried out via a combination of manual journal screening and automated procedures. The database currently contains mutation entries obtained from over 41,000 primary and 15,000 additional (supplementary) literature reports published in more than 1,950 different journals. Of >10,000 identified articles screened for mutation data during 2012, 35 % contained novel mutation data, 29 % contained additional useful information (e.g. in vitro functional data or further clinical or phenotypic information) and were therefore cited as additional references, whilst the remaining 36 % of articles contained no novel mutation data or supporting information to warrant their inclusion as either primary or supplementary references in HGMD. The number of articles screened by HGMD is increasing on a yearly basis; however, we impose no prior limit upon the number of articles we include as supplementary references for a given mutation.

For ~4 % of all the missense/nonsense mutations reported in the literature during 2012, it was necessary for the HGMD Curators to contact the original authors to obtain correction and/or clarification of the nature or precise location of the mutations in question. However, only half of the mutations that required author contact were satisfactorily resolved by these means, leading to their inclusion in HGMD; the ~2 % of unresolved missense/nonsense mutations will not be entered into HGMD unless or until the nature or precise location of the mutation(s) in question is determined to the satisfaction of the HGMD curators. Such data (currently 366 entries) are, however, retained indefinitely by HGMD as part of a “Bad Bank” of inadequately described mutations.

## Classes of variant listed in HGMD

**Fig. 1:**
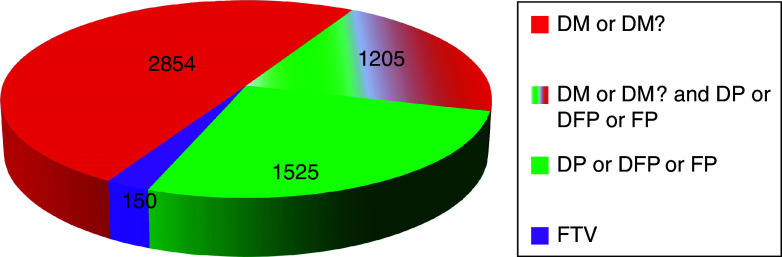
5,734 genes are listed in HGMD professional 2013.2, subdivided here by variant class

**Fig. 2:**
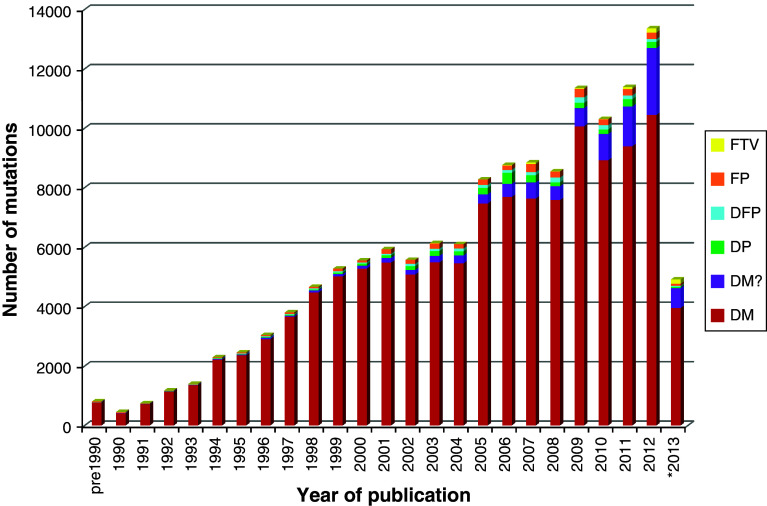
HGMD annual mutation totals subdivided by variant class. *2013 figures to June 28th

There are six different classes of variant present in HGMD (Figs. 1, 2). Disease-causing mutations (DM) are entered into HGMD where the authors of the corresponding report(s) have demonstrated that the reported mutation(s) are involved in conferring the associated clinical phenotype upon the individuals concerned. The DM classification may, however, also appear with a question mark (DM?), denoting a probable/possible pathological mutation, reported to be pathogenic in the corresponding report, but where (1) the author has indicated that there may be some degree of uncertainty; (2) the HGMD curators believe greater interpretational caution is warranted; or (3) subsequent evidence has appeared in the literature which has called the putatively deleterious nature of the variant into question.

Disease-associated polymorphisms (DP) are entered into HGMD where there is evidence for a significant association with a disease/clinical phenotype along with additional evidence that the polymorphism is itself likely to be of functional relevance (e.g. as a consequence of genic/genomic location, evolutionary conservation, transcription factor binding potential, etc.), although there may be no direct evidence (e.g. from an expression study) of a functional effect. Functional polymorphisms (FP) are included in HGMD where the reporting authors have shown that the polymorphism in question exerts a direct functional effect (e.g. by means of an in vitro reporter gene assay or alternatively by protein structure, function or expression studies), but with no disease association reported as yet. Disease-associated polymorphisms with supporting functional evidence (DFP) must meet both of the above criteria in that the polymorphism should not only be reported to be significantly associated with disease but should also display evidence of being of direct functional relevance.

Copy number variations (CNVs) represent an important subset of potentially functional disease-associated variation. While HGMD does not wish to replicate the excellent curatorial work of other resources (e.g. the Database of Genomic Variants http://dgv.tcag.ca/dgv/app/home, DECIPHER http://decipher.sanger.ac.uk/ and Copy Number Variation in Disease http://202.97.205.78/CNVD/), we are nevertheless interested in including such variants (as gross deletions or duplications) if they meet certain criteria. Therefore, HGMD will include such variations if they have been shown to be both of functional significance and associated with disease, and involve a single characterized gene that has itself been directly implicated in the disease association. Such variants would be entered under one of the above-mentioned polymorphism categories, depending upon the supporting evidence provided by the authors of the original reporting article.

In the opinion of the HGMD curators, the polymorphism data present in HGMD should be viewed with a considerable degree of caution owing to (1) the possibility that the observed disease association may be simply due to a linkage disequilibrium effect rather than a bona fide underlying functional mechanism and (2) the fact that in vitro studies are not invariably accurate indicators of in vivo functionality (Cirulli and Goldstein 2007; Dimas et al. 2009).

Finally, frameshift or truncating variants (FTV) are polymorphic or rare variants reported in the literature that are predicted to truncate or otherwise alter the length of the gene product (i.e. a stop-gain, stop-loss or frameshift variant) but with no disease association reported as yet. Most known FTVs have been identified during the course of large-scale genome/exome screening studies (involving either patient panels or apparently healthy individuals from the general population). They may be considered to represent either latent protein deficiencies or, potentially, heterozygous carrier states for recessive disorders. Coverage of FTVs is far from being comprehensive at this juncture, and it remains unclear what proportion will turn out to be clinically significant.

The HGMD curators have adopted a policy of continual reassessment of the curated content within the database. If and when additional and important new information pertaining to a specific mutation entry becomes available (e.g. questionable pathogenicity, confirmed pathogenicity, additional clinical or laboratory phenotypes, population frequency data, supporting functional studies, etc.), then the mutation entry may be revised or even re-categorized. Alternatively, a comment or additional reference may be added in order to communicate this new information to users. Where new information becomes available which suggests that a given disease-causing mutation (DM) is likely to be of questionable pathological relevance or possibly a neutral polymorphism (on the basis of additional case reports, genome/population screening studies, presence in dbSNP with reliable population frequency data, etc.), it may be flagged with a question mark (DM?) or even removed from the database entirely if it turns out to have been erroneously included ab initio. In a recent re-curation exercise, a total of 539 mutations were re-examined due to their presence in the 1000 Genomes Project dataset at a frequency of >1 %. Of the total re-examined, 33 mutations were removed from HGMD, 109 were re-categorized and 220 had additional comments or references added to further justify their inclusion in HGMD (Xue et al. 2012). One reason why some HGMD-listed mutations are often to be found among 1000 Genomes Project data is that many pathogenic lesions are found quite frequently in the population at large (Nishiguchi and Rivolta 2012; Andreasen et al. 2013; Lazarin et al. 2013; Cooper et al. 2013). In addition to internal curation, users of HGMD Professional may utilize a feedback function in order to inform the HGMD curators of relevant new or missing information, to request corrections or to ask for the reclassification or removal of a listed variant.

Most of the clinical phenotypes attributed to DMs in HGMD represent individually rare conditions that are generally regarded as monogenic diseases. However, it is important to note that HGMD also considers a few silent protein deficiencies or biochemical phenotypes (e.g. butrylcholinesterase deficiency, reduced oxygen affinity haemoglobin, etc.) to be worthy of inclusion since they are potentially disease-relevant (even if they are relatively common in the general population); such variants may well be assigned to the DM category.

For individual mutations in HGMD, the provision of zygosity information (heterozygous, homozygous or compound heterozygous) has not been attempted. Reasons for this include (1) the fact that this information is not always unambiguously provided in the corresponding article; (2) the possibility that a given mutation may be pathogenic irrespective of the zygosity in which it is found; (3) the clinical consequences of zygosity may often be modified by other genetic variants either in *cis* or in *trans* and (4) the general phenomenon of variable or reduced penetrance which ensures that the genotype is not invariably predictive of the phenotype (Cooper et al. 2013). Thus, information pertaining to zygosity would not always be helpful or informative with regard to ascertaining or predicting the clinical phenotype, and indeed might even prove inaccurate or misleading.

HGMD users should not assume that just because a mutation is labelled “DM”, that it automatically follows that the mutation is known or believed to be pathogenic in all individuals harbouring it (i.e. that the mutation exhibits 100 % penetrance). This is not invariably going to be the case and many “disease-causing mutations” will display reduced or variable penetrance for a variety of different reasons (reviewed by Cooper et al. 2013). Indeed, next generation sequencing programmes (such as the 1000 Genomes Project) are now identifying considerable numbers of “DM” mutations in apparently healthy individuals (MacArthur et al. 2012; Xue et al. 2012). Such lesions should not automatically be regarded as being clinically irrelevant because it is quite possible that they represent low-penetrance, mild or late onset, or more complex disease susceptibility alleles, as opposed to neutral variants (Cooper et al. 2013).

It has always been HGMD policy to enter a variant into the database even if its pathological relevance may be questionable (while indicating this fact wherever feasible to our users), rather than run the risk of inadvertently excluding a variant that may be directly (or indirectly) relevant to disease. We have taken several steps to highlight such equivocation in HGMD, viz. the recent introduction of the DM? variant class, a dbSNP 1000 Genomes frequency flag (to highlight HGMD variants that are also present in dbSNP, with allele frequency information included; see below) and the provision of additional literature citations where the pathogenicity of the variant may have been subsequently either questioned or confirmed. This latter point is particularly pertinent in the clinical setting, where a greater burden of proof may be required for use in diagnostic and predictive medicine, and when considering the return of incidental findings to patients after testing (Green et al. 2012, 2013; Ng et al. 2013; Gonsalves et al. 2013).

## HGMD Professional

HGMD Professional has been developed to serve as the subscription version of HGMD, and is available to both commercial and academic customers under license from BIOBASE GmbH. HGMD Professional allows access to up-to-date mutation data with a quarterly release cycle; this version is therefore essential for checking the novelty of newly found mutations. HGMD Professional contains many features not available in the free public version (Table 2). More powerful search tools in the form of an expanded search engine with full text Boolean searching are provided. A batch search mode has recently been developed to allow users to search HGMD using gene (e.g. OMIM IDs) and variant (e.g. dbSNP IDs) oriented lists. Users can employ these tools to perform additional searches for gene-specific (e.g. chromosomal locations, gene names/aliases and gene ontology), mutation-specific (e.g. chromosomal coordinates, HGVS nomenclature, dbSNP ID) or citation-specific (e.g. first author, publication year, PubMed ID) information. The provision of chromosomal coordinates (hg19) for the vast majority of our nucleotide substitutions (98.7 % coverage) and other micro-lesions (97.3 % coverage) has made HGMD an invaluable tool for the large-scale analysis of NGS datasets such as the 1000 Genomes Project (1000 Genomes Project Consortium 2010, 2012). Additional information is also provided on a mutation-specific basis including curatorial comments pertaining to particular mutations (for example, if the mutation data presented required in-house correction in relation to the data presented in the original publication [5–10 % of entries], or if the clinical phenotype is associated with a more complex, i.e. a digenic or SNP in-*cis* inheritance pattern), additional reports comprising functional characterisation, further phenotypic information, comparative biochemical parameters, evolutionary conservation and SIFT (Sim et al. 2012) and MutPred (Li et al., 2009) predictions. These additional annotations are updated on a regular basis.

Recently, HGMD clinical phenotypes have been annotated against the Unified Medical Language System (UMLS) using a combination of manual curation and natural language processing. The UMLS is a comprehensive collection of biomedical concepts and the relationships between them (http://www.nlm.nih.gov/research/umls/). These UMLS mappings provide users with a more accurate and expanded phenotype search. Thus, searches using alternative disease names will return the same result-set, e.g. a search for “breast cancer” would yield identical results to a search for “malignant breast tumour”. In addition, utilizing the UMLS allows for powerful semantic searching (e.g. searches for all mutations linked to blood disorders or all immune disorders).

Another new feature involves the highlighting of HGMD entries where the pathogenicity of the variant may have been cast into doubt by virtue of its allele frequency. HGMD Professional now displays a frequency flag when a listed variant is also found in dbSNP, and population frequency data from the 1000 Genomes Project are also provided. HGMD data have also recently been made available in Variant Call Format or VCF (Danecek et al. 2011), which will facilitate the comparison of HGMD with large NGS datasets. In addition to searching and viewing mutation data in a variety of ways, users of HGMD Professional may utilize a new feedback facility to submit corrections to the database curators or to request additional features.

**Fig. 3:**
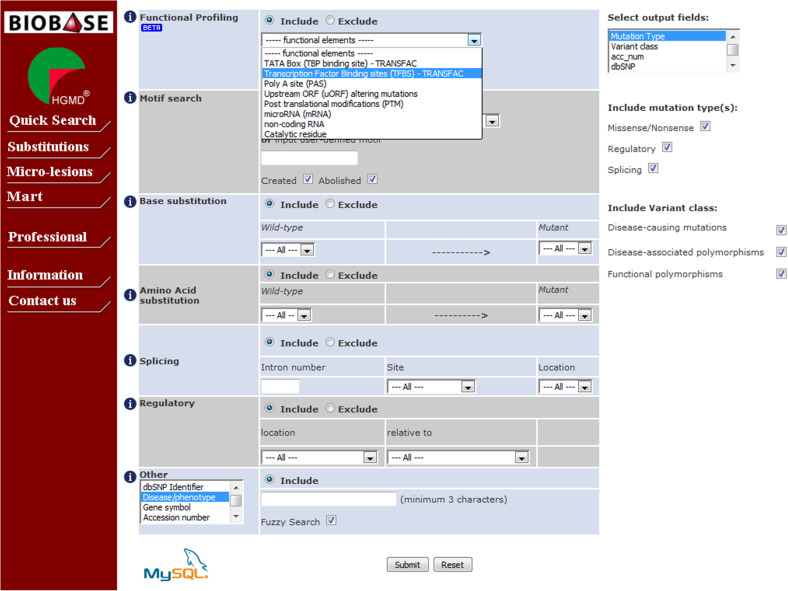
Advanced nucleotide substitutions search in HGMD Professional

HGMD Professional also contains an Advanced Search suite which has been designed to enhance mutation searching, viewing and retrieval. Two of the main types of mutation in HGMD (single-nucleotide substitutions and micro-lesions) can be interrogated with this toolset. Datasets for more than one mutation type may be combined (for example, micro-deletions, micro-insertions and indels) to enable more powerful searching across comparable types of mutation. When using the Advanced Search, users can tailor their queries with more specific criteria, including functional profile (e.g. in vitro and in silico characterized transcription factor binding sites, post-translational modifications, microRNA binding sites, upstream ORFs, and catalytic residues, see Fig. 3); amino-acid change; nucleotide substitution; size and/or sequence composition of micro-deletions, micro-insertions or indels; pre- or user-defined sequence motifs (both those created and those abolished by the mutation); dbSNP number; keywords found in the article title or abstract. Results returned by the Advanced Search can be downloaded as tab-delimited text or a genome browser track, ready to be used in different applications. The Advanced Search also includes a batch mode called “Mutation Mart” to query HGMD via multiple identifiers including dbSNP, Entrez gene (http://www.ncbi.nlm.nih.gov/gene) and PubMed. HGMD Professional is available to subscribers either as an online only package or in downloadable form enabling users to incorporate HGMD data into their local variant analysis pipelines (http://www.biobase-international.com).

## Other variant databases

Several other databases are available that attempt to record disease-causing or disease-associated (i.e. pathogenic) variation. These include the Online Mendelian Inheritance in Man, OMIM (http://www.omim.org/; Amberger et al. 2009), ClinVar (http://www.ncbi.nlm.nih.gov/clinvar/), dbSNP (http://www.ncbi.nlm.nih.gov/SNP/; Sherry et al. 2001) and an assorted collection of locus-specific mutation databases (LSDBs) (http://www.hgvs.org/dblist/glsdb.html). OMIM does not provide statistics for allelic variants on its website; however, 22,901 germline OMIM variants appear to have been added to ClinVar, which itself contains a total of 25,375 pathogenic and probable pathogenic germline variants, while dbSNP contains 23,973 pathogenic or probable pathogenic germline variants (all databases were accessed July 10th 2013). Owing to the highly dispersed nature of the LSDBs and the potential for duplication between databases, accurate statistics with regard to like-for-like bona fide germline disease-causing (not merely neutral) variation is difficult to obtain. Since OMIM only records a limited number of variants per gene, and ClinVar is still in its infancy, HGMD is the only database of human pathological mutations that approaches comprehensive coverage of the peer-reviewed literature (Peterson et al. 2013). Since ClinVar and the LSDBs contain unpublished (non-peer reviewed) mutation data, the question has arisen as to whether HGMD should also include these data (Patrinos et al. 2012). However, several obstacles have been encountered by the LSDBs, including serious problems pertaining to data quality as well as issues of data provenance and consent. HGMD has therefore taken the decision not to include such data at this time.

## How HGMD is utilized

Registered users of the public HGMD website currently number in excess of 60,000. Users may not download HGMD data in their entirety. However, mutation data may be made available at the discretion of the curators for non-commercial research purposes. Potential collaborators who wish to access HGMD data in full are required to sign a confidentiality agreement.

HGMD data have been used to perform an extensive series of meta-analyses on different types of gene mutation causing human inherited disease. These studies have helped to improve our understanding of mutational spectra and the molecular mechanisms underlying human inherited disease (Cooper et al. 2011). They have served to demonstrate not only that human gene mutation is an inherently non-random process but also that the nature, location and frequency of different types of mutation are shaped in large part by the local DNA sequence environment (Cooper et al. 2011). HGMD data have been used extensively in several international collaborative research projects including the 1000 Genomes Project (1000 Genomes Project 2010, 2012), where a surprising number of HGMD variants were found in apparently healthy individuals. They have also been used in the comparative analysis of several orthologous genomes including gorilla (Scally et al. 2012), cynomolgus and Chinese macaque (Yan et al. 2011), Rhesus macaque (Rhesus Macaque Genome Sequencing and Analysis Consortium 2007) and rat (Rat Genome Sequencing Project Consortium 2004), in which many apparently disease-causing mutations in human were found as wild type (‘compensated mutations’).

In a clinical setting, HGMD is widely utilized by many groups in ongoing NGS diagnostic (Johnston et al. 2012, Calvo et al. 2012, Bell et al. 2011) and human genome sequencing (Tong et al. 2010; Kim et al. 2009) programmes. HGMD has also been used by a number of different groups to aid the development of post-NGS variant interpretation algorithms including MutPred (Li et al. 2009), PROVEAN (Choi et al. 2012), CAROL (Lopes et al. 2012), CRAVAT (Douville et al. 2013), NEST (Carter et al. 2013) and FATHMM (Shihab et al. 2013). Finally, HGMD has been used as a resource for structural biologists in the reconstruction of protein interaction networks (Wang et al. 2012; Guo et al. 2013). A more complete list of articles which have utilized HGMD data or expertise in their production can be found on the HGMD website (http://www.hgmd.cf.ac.uk/docs/articles.html).

## Data sharing

A limited HGMD data set, containing both chromosomal coordinates and HGMD identifiers, has been made available via academic data exchange programmes to the Gen2Phen project (Webb et al. 2011), the European Bioinformatics Institute (EBI)/Ensembl (Flicek et al. 2013) and the University of California, Santa Cruz (UCSC) (Meyer et al. 2013) and may be viewed in these projects’ respective genome browsers. Data from HGMD Professional have additionally been made available to HGMD subscribers via Genome Trax™ (BIOBASE GmbH) and Alamut (Interactive Biosoftware), but are also accessible as part of the HGMD Professional stand-alone package (BIOBASE GmbH). Allowing free access to the bulk of the mutation data present in HGMD, while generating sufficient income from its commercial distribution to support its maintenance and expansion, represents a business model that should maximize the availability of HGMD at the same time as ensuring its long-term sustainability. Although we are necessarily obliged to be prudent with regard to data sharing with public data repositories, we have always taken the view that making as much data as possible publicly available is generally beneficial to both HGMD and its users worldwide.

## Future plans

The provision of chromosomal coordinates for the vast majority of coding region micro-lesions in HGMD is now complete. Expanding this provision to include micro-lesions in non-coding regions and the gross and complex lesion dataset (where feasible) is a high priority, as is expanding the provision of genomic coordinates to include popularly utilized NGS formats such as General Feature Format (GFF) (http://www.sanger.ac.uk/resources/software/gff/) and BED format, to complement the recently added HGMD Variant Call Format (VCF) (Danecek et al. 2011). Mutations will also be mapped to the new genome build (GRCh38) in due course. A listing of removed variants will be implemented as time allows. Provision of genomic reference sequences based on the NCBI RefSeqGene project (Pruitt et al. 2009), links to available protein structures and homology models, and mapping HGMD phenotypes to the Human Phenotype Ontology (HPO) are also regarded as priorities.

In its current state of development, HGMD provides the user with a unique resource that can be utilized not only to obtain evidence to support the pathological authenticity and/or novelty of detected gene lesions and to acquire an overview of the mutational spectra for specific genes, but also as a knowledgebase for use in the bioinformatics and whole genome screening projects that underpin personalized genomics.
